# Chicago Medical Society

**Published:** 1872-04-01

**Authors:** 


### CHICAGO MEDICAL SOCIETY.

Regular meeting, April 11th. G. C. Poole, President, in the Chair.

After the usual preliminary business, the subject of cerebro-spinal meningitis was introduced by a short paper, read by Dr. N. S. Davis. (See first article in the present number of EXAMINER.) After the reading of the paper, Dr. E. Powell stated that he had recently witnessed two post-mortem examinations of patients who had died from well-marked symptoms of this disease. The first was a patient who died on the third day after admission into the county hospital. A moderate amount of bloody serum was found between the arachnoid and pia-mater, with a thin layer of plastic exudation over the base of the brain. The blood vessels were also minutely filled with blood. The second case was in the West Division of the city, and presented the same morbid appearance; except there was no plastic exudation.

Dr. Ingalls, of the county hospital, read an interesting account of six cases that had been admitted and treated in that institution. Of these five recovered and one died. The remedy that was chiefly relied on, and which appeared to produce a very favorable effect, in the treatment of those cases, was the tincture of cannabis indica. Its exhibition was generally commenced in doses of 20 minims every two hours, and rapidly increased to a fluid drachm, unless the symptoms soon began to abate.

Dr. N. Loverin referred to two cases, both of which had proved fatal in a short time. He expressed more confidence in the efficacy of the early abstraction of blood by venesection, followed by narcotics.

Dr. Marguerat had met with six or eight cases, but had found no plan of treatment productive of satisfactory results. To one child, presenting the initial symptoms of the disease, aged 18 months, he had given 10 grs. of sulphate of quinia per day, with a rapid recovery. Another child, aged two years, had recovered while under the active influence of quinia.

Dr. T. D. Fitch had treated only two cases that he regarded as cerebro-spinal meningitis. One of these had recovered and the other was still under treatment. He had given them the bromide and iodide of potassa, and the tincture of calabar bean, with fluid extract of lettuce, with apparent benefit.

Dr. S. Wickersham referred to the disease as it prevailed in 1859–60, comparing it with the present epidemic. He thought the disease in the former years presented more constant muscular rigidity and cutaneous spots than at the present time, and less frequent vomiting or gastric disturbances. The first marked case he had met with this year was in the present month (April). He found hydrate of chloral at first to produce no apparent relief; but he gave veratrum viride in sedative doses, and as soon as the pulse was reduced in frequency to near the normal standard, the chloral acted more favorably and induced quiet sleep, followed by rapid improvement. He thought the chloral would be found generally to act favorably, if its use was preceded by the sedative influence of veratrum viride.

Dr. C. W. Earle referred to the severe prevalence of the disease in some parts of Lake county, in this State, when the further discussion of the subject was postponed until the next regular meeting of the society, which will be on the first Monday evening in May.

